# Antioxidant Strategies for Age-Related Oxidative Damage in Dogs

**DOI:** 10.3390/vetsci12100962

**Published:** 2025-10-09

**Authors:** Aljaž Muršec, Borut Poljšak, Alenka Nemec Svete, Vladimira Erjavec

**Affiliations:** 1 Laboratory of Oxidative Stress Research, Faculty of Health Sciences, University of Ljubljana, 1000 Ljubljana, Slovenia; aljaz.mursec@zf.uni-lj.si (A.M.); borut.poljsak@zf.uni-lj.si (B.P.); 2 Small Animal Clinic, Veterinary Faculty, University of Ljubljana, 1000 Ljubljana, Slovenia; alenka.nemecsvete@vf.uni-lj.si

**Keywords:** oxidative stress, antioxidants, aging, dogs, cognitive dysfunction

## Abstract

**Simple Summary:**

This paper explores how oxidative stress affects aging in dogs and how antioxidants can help. As dogs get older, their bodies produce more harmful molecules called free radicals, which can damage cells and lead to problems like memory loss, chronic illness, and a lower quality of life. Antioxidants—such as vitamins C and E, coenzyme Q_10_, and plant compounds called polyphenols—can help protect dogs by neutralizing these harmful molecules. Research shows that giving dogs a balanced diet with added antioxidants may improve their health and reduce the risk of age-related diseases, contributing to heathier aging.

**Abstract:**

This review examines the effects of oxidative stress on the aging process in canines, focusing on the role of antioxidants in the prevention of age-related diseases. Oxidative stress is caused by an imbalance between the production of free radicals and the body’s antioxidant defenses, resulting in damage to cell structures. Dogs, especially older animals, are particularly susceptible to such damage, which contributes to the development of cognitive impairment, chronic disease and a reduced quality of life. Antioxidants such as vitamins C and E, coenzyme Q_10_ and polyphenols play an important role in neutralizing free radicals and mitigating oxidative damage. Various studies confirm that these antioxidants can improve overall health, slow cognitive decline and reduce the risk of diseases such as osteoarthritis, cancer and heart disease. The results suggest that an appropriate diet supplemented with antioxidants can significantly contribute to a better quality of life for dogs. However, given that some studies report limited or no effects, additional long-term clinical trials are warranted to validate the reproducibility and degree of presented benefits.

## 1. Introduction

**Figure 1:**
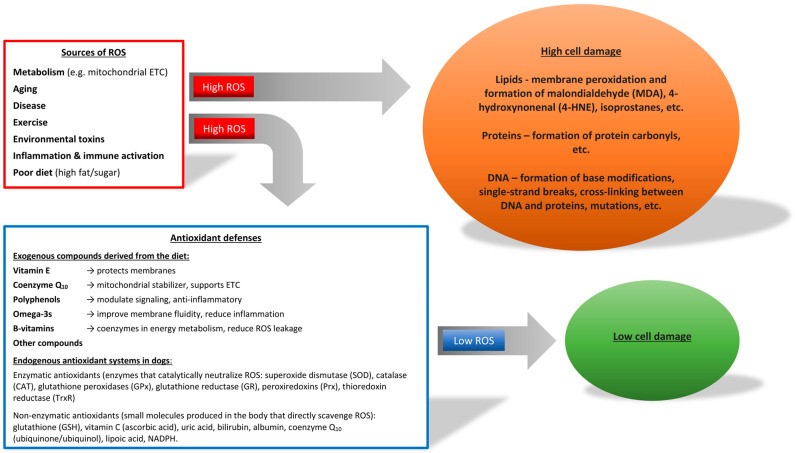
Oxidative Stress Pathways and Antioxidant Defense Mechanisms. ETC = Electron Transport Chain; ROS = Reactive Oxygen Species.

Reactive oxygen and nitrogen species are generated by endogenous and exogenous processes, while enzymatic and non-enzymatic antioxidants neutralize them. An imbalance between reactive oxygen species (ROS) production and antioxidant defenses leads to oxidative stress (Figure 1), causing disrupted redox signaling or molecular damage [1,2,3]. External contributors include pollutants, pesticides, radiation, lifestyle, medications, and toxins; internal sources are mainly the mitochondrial electron transport chain, chronic inflammation, and impaired antioxidant defenses (e.g., altered NADPH enzyme activity) [4]. Oxidative stress disrupts tissue and organ function, ultimately causing systemic dysfunction [5]. Cell membranes are especially vulnerable due to lipid peroxidation, which alters permeability; its by-products, along with damaged proteins and DNA, can be measured in tissues and fluids [6,7]. Such damage contributes to degenerative diseases including cancer [8], cardiovascular disorders [9], and aging [1,4]. It also impairs membrane transport, as in galactosemia [10,11,12], and occurs during infections (e.g., Plasmodium falciparum), contributing to pathogenesis [13,14]. Neurons are particularly sensitive, with mitochondrial damage leading to neurodegeneration and cell death in Alzheimer’s, Parkinson’s, and epilepsy [15,16]. Normally, protection relies on both exogenous antioxidants (diet, supplements, drugs) and endogenous systems (metabolic pathways) [17,18]. Enzymatic defenses include superoxide dismutase (SOD), catalase, and glutathione peroxidase, while non-enzymatic agents (glutathione, albumin, uric acid, coenzyme Q_10_) scavenge free radicals [17,19,20]. If these fail, repair systems restore oxidized DNA, proteins, and membranes [21].

Elevated oxidative stress is typical in metabolically active tissues such as muscle, liver, and blood [22,23]. In animals, it is induced by environmental, physical, chemical, biological, and psychological stressors; for example, heat reduces productivity, and behavioral stress impairs immunity and fertility [24]. Oxidative stress is linked to numerous diseases in human and veterinary medicine [25]. In dogs, it has been documented in anesthesia [26], heart failure [27,28,29,30,31], anemia [32,33], atopic dermatitis [34,35,36], cancer [37], spinal cord injury [38], inflammatory bowel disease [39], and infections [40,41,42,43,44]. Biomarker levels vary due to methodological and preanalytical issues and are influenced by breed [45,46], age [45,47,48], sex [48,49], neuter status, and diet [49,50]. Free radicals and reactive oxygen and nitrogen species are responsible for cellular damage in the body. Nutritional intake plays an important role in mitigating this damage: on the one hand, the presence of essential fatty acids in membrane composition reduces cellular susceptibility to free radicals and serves as a primary defense against oxidative stress [51,52]; on the other hand, antioxidant molecules [17] such as CoQ_10_, vitamin E, vitamin C, and polyphenols help neutralize free radicals within cells [27]. However, an unhealthy diet, particularly one high in saturated fats, refined sugars, and processed foods, has been shown to promote oxidative stress by increasing the production of ROS and impairing antioxidant defense systems. Excessive caloric intake and poor nutrient composition can lead to mitochondrial dysfunction, chronic inflammation, and reduced levels of endogenous antioxidants such as glutathione [53,54]. For example, high-fat diets have been associated with increased lipid peroxidation and oxidative damage to cellular components [55]. Moreover, diets low in antioxidant-rich foods—such as fruits, vegetables, and whole grains—fail to provide sufficient exogenous antioxidants, further exacerbating oxidative imbalance [52,56].

The aim of this review is to thoroughly investigate the impact of oxidative stress on canine aging, with a particular focus on the role of antioxidants and their potential benefits in combating aging and preventing pathological processes such as chronic diseases. In recent years, several studies have demonstrated that oxidative stress is one of the key contributors to cellular aging, including in dogs. Therefore, it is crucial to understand whether and how antioxidants—known for their ability to reduce oxidative stress—can influence the maintenance of health in canines.

Through this analysis, we aim to contribute to a deeper understanding of the oxidative mechanisms underlying aging in dogs and to emphasize the importance of preventive measures, such as appropriate antioxidant-rich nutrition, in preserving the health and vitality of canines in their senior years.

## 2. Materials and Methods

In this study, a comprehensive literature search was performed across four major academic databases (Google Scholar, Web of Science, ScienceDirect, and PubMed) with the aim of identifying publications relevant to the themes of “dogs/canids” and “oxidative stress/antioxidants/aging”. These core search terms were combined using the Boolean operators OR and AND to maximize retrieval of pertinent studies. The search was restricted to peer-reviewed scientific literature, and the final inclusion criteria required that the selected publications directly addressed the specific focus of this review.

## 3. The Correlation Between Oxidative Stress and Aging in Canids

**Figure 2:**
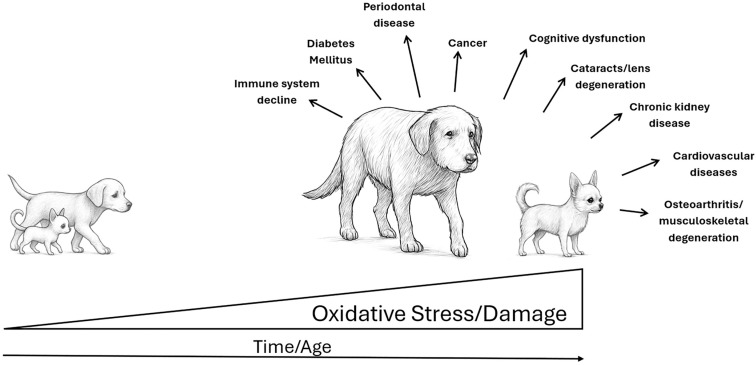
Age- and breed-related increases in oxidative stress contributing to canine degenerative conditions.

Aging is a natural process accompanied by numerous physiological changes. Older dogs are more likely to develop various age-related diseases [57]. Canids represent one of the most diverse groups of mammals, with domestic dogs exhibiting the greatest phenotypic plasticity in terms of body size, coat length, coloration, and limb morphology [58,59,60,61]. Generally, smaller dogs live longer than larger breeds and often have a lower risk of certain age-related conditions, such as cognitive dysfunction, cancer and cardiovascular diseases (Figure 2). However, some aging signs, including cataracts, are commonly observed across dogs of all sizes and breeds [58,62,63]. This inverse relationship between body size and lifespan is unique to domestic dogs and is thought to result from accelerated growth rates and higher metabolic demands in large breeds, which may increase oxidative damage and cellular damage [64]. Large dogs also have faster rates of cellular proliferation, which may elevate the risk of cancer through more frequent DNA replication errors [65]. In contrast, smaller breeds often experience slower aging and accumulate fewer age-associated pathologies, including lens degeneration and cataracts, likely due to reduced systemic inflammation and better mitochondrial efficiency [58,66].

Research has shown that also the insulin-like growth factor 1 (IGF-1) haplotype plays a critical role in determining size differences in dogs, with smaller breeds exhibiting lower levels of IGF-1, which may contribute to their increased longevity [67]. In a study by Sutter et al. (2007) [68], a single nucleotide polymorphism deoxyribonucleic acid in the IGF-1 gene haplotype was found in all small breeds and was almost entirely absent in large breeds. This suggests that the same genetic variant exerts a strong influence on body size across small dogs. Paradoxically, smaller breeds tend to have higher metabolic and growth rates than larger ones, which predisposes them to higher levels of ROS production [63,66,67,69]. Interestingly, also some other animal species—such as vampire bats, naked mole rats, and certain birds—display higher levels of oxidative DNA damage compared to their relatives (e.g., house mice), yet still achieve considerably greater longevity [70]. This implies that the production of ROS alone does not determine lifespan to the same extent as the degree of oxidative damage sustained by cells. If sufficient endogenous and exogenous antioxidants are available, oxidative damage can be prevented; however, effective cellular repair systems are also essential to remove and restore biomolecules, such as DNA, proteins, and lipids, that may already have been oxidatively modified [17,21]. Moreover, mild ROS exposure may induce a hormetic effect, enhancing endogenous defence mechanisms through multiple signalling pathways thus decreasing oxidative stress induced damage by enhancing its repair [71,72,73,74].

Notably, wild canids such as wolves—close relatives of domestic dogs—tend to live longer than domestic dogs of comparable size. For instance, the grey wolf, the closest ancestor of domestic dogs, can live up to 20 years, whereas a similarly sized breed such as the Cane Corso typically has a lifespan of only 10–12 years. This observation suggests that key physiological and cellular differences may exist between domestic dogs and their wild relatives, influencing longevity [75]. Oxidative stress may be one of the contributing factors to these differences [75,76]. In a study conducted by Jimenez and Downs (2020) [75], the authors measured oxidative stress parameters either in plasma or in erythrocytes (depending on a parameter) from domestic dogs and wild canids of various ages and body sizes. They found that patterns of oxidative stress differed between the two groups, potentially contributing to differences in aging processes related to body size. The study showed that lipid damage increased with age in domestic dogs, whereas large wild canids such as grey wolves exhibited enhanced antioxidant defences as age and body mass increased. This could explain their extended longevity and highlight important physiological differences within the canid family. In small-breed dogs, significantly higher levels of lipid peroxidation were observed compared to larger breeds. These findings suggest that artificial selection in domestic dogs may have inadvertently reduced antioxidant capacity and that small dogs may survive longer despite experiencing increased oxidative damage. However, the authors noted that blood samples from wild canids were collected in a zoological setting. This raises the question of whether the findings are fully representative or generalizable to wild-living canids that do not receive veterinary care, such as vaccinations, antiparasitic treatments, and regular, balanced nutrition. It is conceivable that results might differ if wild canids in natural habitats were included in the sample [75].

Taken together, these studies show both convergence and divergence in the relationship between oxidative stress, body size, and longevity in canids. According to studies, there is consistent evidence that smaller dogs live longer, partly due to genetic factors such as IGF-1 and differences in mitochondrial efficiency. At the same time, there are paradoxes: small dogs often produce more ROS yet still accumulate fewer pathologies, while wild canids of comparable size live longer than domestic breeds, suggesting that selective breeding has altered antioxidant capacity. The overall evidence indicates that the lifespan in canids is shaped not simply by ROS production, but by the balance between oxidative damage, antioxidant defenses, and repair mechanisms, with artificial selection introducing additional complexity compared to wild relatives.

## 4. The Impact of Oxidative Stress on Canine Cognitive Dysfunction and the Role of Antioxidants

The deterioration of physical or cognitive health in aging dogs can be challenging for owners, as behavioral and cognitive changes in their pets may strain the human–animal bond. While many owners often perceive these changes as a normal part of aging, it is crucial to distinguish between physiological aging and pathological processes. Behavioral changes can serve as early indicators of declining health and well-being in senior dogs [77]. Cognitive decline in aging dogs may have a biological basis, and many age-related disorders can be mitigated with nutritional supplements containing antioxidants [78,79]. Antioxidants protect cells by neutralizing free radicals by donating electrons to stabilize free radicals without becoming reactive themselves, effectively halting the chain reactions that lead to oxidative damage [15]. For example, antioxidants as supplements have been shown to exert beneficial effects in elderly dogs [51,80]. The brains of older dogs accumulate oxidative damage to proteins and lipids, potentially leading to neuronal dysfunction [52,78,79,80]. Reducing oxidative damage through antioxidant-rich dietary components significantly improves or slows the decline in learning and memory in senior dogs [52,78,79,80,81,82]. Research has shown that oxidative stress is a significant factor in brain aging and the development of cognitive dysfunction in dogs. Older dogs exhibit neurocognitive behavioral changes that are attributed to increased oxidative damage in the brain, suggesting that oxidative stress plays a role in brain aging and cognitive decline [78,79]. Similarly to humans, brain aging in dogs is exposed to risk factors such as deficiencies in specific nutrients (e.g., docosahexaenoic acid (DHA); vitamins B6 and B12; and folic acid), increased oxidative stress and inflammation, as well as diseases such as hypertension, diabetes, and obesity [83,84,85]. These factors gradually impact the brain and can contribute to cognitive decline, analogous to Alzheimer’s disease in humans [86,87,88].

How does oxidative stress influence the brain aging in dogs? Damaged mitochondria produce abnormally high levels of ROS [89], which contribute to the deterioration of cognitive function and interfere with normal cellular processes in neurons [78,90]. Mitochondria are thus the main site of intracellular oxygen consumption and the main source of ROS formation [91,92]. Briefly, healthy mitochondria generate adenosine triphosphate (ATP) through oxidative phosphorylation. During this process, a small proportion of the electrons “escape” from the electron transport chain (ETC) and react with oxygen to form ROS. At low levels, the ROS are largely neutralised by mitochondrial antioxidants (such as Mn-SOD, glutathione) so that no significant damage occurs. When ROS production increases (due to ageing, microbial infections, extensive exercise, or pollutants/toxins [93] such ionizing and UV radiation, pesticides, and ozone, etc.) and antioxidant defences are overwhelmed, ROS can damage mitochondrial lipids (especially cardiolipin in the inner membrane) and mitochondrial DNA and inactivate ETC proteins. This damage impairs the flow of electrons in the ETC. Estimates of how much oxygen reacts directly to generate free radicals vary; however, typically cited values are around 1.5–5% of the total consumed oxygen [94,95]. These estimates have been questioned by Hansford et al. (1997) [96] and Staniek and Nohl (1999) [97] who suggested that H_2_O_2_ production rates were less than 1% of consumed O_2_. This self-repeating cycle, also known as the vicious circle of ROS and mitochondrial damage, can eventually lead to mitochondrial dysfunction, cell death and tissue damage. Namely, oxidative damage may represent a key factor driving neuronal impairment and the gradual development of neuropathological changes observed in older animals [78]. Dietary antioxidants can neutralize ROS (Figure 2), particularly those that damage mitochondria, thereby reducing the accumulation of mitochondrial damage and the risk of cognitive dysfunction [98]. Antioxidants can mitigate the cellular toxicity of ROS by neutralizing them, thereby interrupting oxidative stress–mediated pathways involved in disease pathogenesis [52,78,99]. In a study by Cotman et al. (2002) [79], a diet containing antioxidants and mitochondrial cofactors—key to maintaining cellular energy efficiency and protecting against oxidative stress—was used. These included vitamins E and C, spinach, tomatoes, grapes, carrots, citrus fruits, alpha-lipoic acid, and L-carnitine. In the study, 48 older and 17 younger beagle dogs were fed either an antioxidant-enriched diet (supplemented with dried fruits and vegetables) or a standard diet. After six months, senior dogs on the antioxidant diet made fewer errors on complex tasks compared to the control group. Improvements in spatial orientation and visual discrimination were observed, alongside reduced oxidative damage and decreased accumulation of amyloid-beta plaques in the brain. However, it did not prevent neuronal loss in the hippocampus, suggesting that while antioxidants can improve cognition, they may not halt irreversible neuronal degeneration.

Various studies testing combinations of nutrients and bioactive compounds have shown improvements in learning and memory in aging dogs. One such nutritional blend included antioxidants, B vitamins, DHA/ eicosapentaenoic acid (EPA), and arginine, which successfully slowed age-related cognitive decline in both dogs and cats [86].

In a field-based behavioral study [80], dogs over seven years of age showing signs of cognitive dysfunction were enrolled. The test group (*n* = 61) received an antioxidant-enriched diet (vitamins E and C, DHA, EPA, lipoic acid, L-carnitine, dried fruits, and vegetables), while the control group (*n* = 64) received standard food. After 60 days, the dogs on the enriched diet showed improvements in all categories: environmental awareness, recognition and interaction with family and other animals, spatial orientation, and sleep quality. These dogs were more alert, more socially interactive, less prone to indoor accidents, and more active. In the control group, 27% of dogs showed improvement, compared to 87% in the test group [80].

Studies confirm a link between the immune system and age-related changes in the central nervous system in dogs [89,100]. Aging alters immune function, including changes in the activity of various components within the brain [101]. Alterations in inflammatory markers such as interleukin-6 (IL-6) and C-reactive protein are associated with cognitive decline. This contributes to oxidative stress and damage to the nervous system. A lack of antioxidants and elevated oxidative stress are key factors in the aging process and the development of neurodegenerative diseases [102,103,104].

Overall, studies consistently converge on the idea that oxidative stress is a key driver of canine cognitive dysfunction, mirroring mechanisms described in human neurodegenerative disease. Across multiple investigations, antioxidant-rich diets and supplements have been shown to improve learning, memory, and behavior in aging dogs, with particular benefits for spatial orientation, environmental awareness, and social interaction. However, findings also diverge in their scope: while some interventions reduce oxidative damage and improve cognitive performance, they do not always prevent neuronal loss or halt the progression of neurodegeneration, indicating that antioxidants may be more effective at slowing functional decline than reversing structural brain changes. Together, this body of evidence suggests that oxidative stress contributes substantially to brain aging in dogs, but that nutritional interventions work best when used as preventive or early therapeutic strategies rather than as stand-alone cures for advanced disease.

## 5. The Impact of Nutrition on Oxidative Stress in Dogs

The impact of nutrition on oxidative stress in dogs remains a poorly researched scientific area; nonetheless some key findings related to this topic are presented in this section and were introduced in previous section.

Pathological and physiological changes associated with age-related, exercise-related or disease-related oxidative stress can be mitigated through appropriate nutritional strategies. Adjusting nutrient intake or modifying dietary approaches may be beneficial; however, this is most effectively implemented within the framework of an individually tailored nutritional assessment and close monitoring [57,105,106]. Nutrition plays a crucial role in managing oxidative stress in dogs, as appropriately selected nutrients can help reduce oxidative damage in the body [51,107,108]. Diets that include high-quality protein sources, essential fatty acids, and antioxidant vitamins and minerals can contribute to improved health and longevity in dogs by maintaining cellular balance and reducing the harmful effects of oxidative stress [108,109,110]. These nutrients may also help reduce inflammation and positively influence conditions such as osteoarthritis, kidney disease, cancer, and others [111]. Medium-chain triglycerides show promise in managing canine cognitive dysfunction, as they are metabolized into ketone bodies that can serve as an alternative energy source for the central nervous system [112].

Omega-3 fatty acids are important for protecting cells from oxidative stress. Fatty acids are classified based on the number of double bonds and may be saturated, monounsaturated, or polyunsaturated. Omega-3 polyunsaturated fatty acids, such as EPA and DHA, are highly prone to peroxidation due to their multiple double bonds. Paradoxically, however, omega-3s have been shown to reduce oxidative stress in vivo [113,114]. This protective effect is attributed to several mechanisms, such as the regulation of antioxidant defenses [51,115], anti-inflammatory action, and their membrane effects and mitochondrial function as omega-3s incorporate into cellular membranes, improving fluidity and function [116,117]. For example, omega-3 fatty acids have been shown to reduce oxidative stress in vivo by enhancing endogenous antioxidant defenses, as evidenced by increased total antioxidant capacity and glutathione peroxidase activity, together with decreased lipid peroxidation markers malondialdehyde (MDA) in clinical trials [115]. Essential fatty acids, such as omega-3 and omega-6, must be obtained through the diet because the body cannot synthesize them. Oxidative stress primarily damages the unsaturated fatty acids in cell membranes, leading to cellular dysfunction. Essential fatty acids reduce the susceptibility of cellular membranes to free radicals, thus protecting cells from oxidative stress. In a study on rats, supplementation with omega-3 fatty acids increased levels of endogenous antioxidants, such as SOD, which neutralizes free radicals in heart cells was reported [118,119,120]. The study by Kearns et al. (1999) investigated the effects of age, breed, and dietary omega-6/omega-3 fatty acid ratio on immune function and oxidative stress in young and aged dogs [121]. Reducing the ratio from 25:1 to 5:1 enhanced T- and B-cell responsiveness, increased production of Prostaglandin E3 without affecting pro-inflammatory cytokines or Prostaglandin E2, and lowered MDA levels in older dogs, indicating reduced lipid peroxidation. No changes were observed in 4-Hydroxynonenal or vitamin E, although aged animals consistently had lower vitamin E concentrations [27,121].

In addition to fatty acids, antioxidants such as CoQ_10_ are important for neutralizing free radicals. CoQ_10_ is an endogenous lipophilic molecule in the inner mitochondrial membrane. It facilitates electron transport from Complexes I and II to Complex III, a critical part of ROS formation and ATP synthesis [122,123,124]. CoQ_10_ is synthesized endogenously from amino acids (tyrosine/phenylalanine) and via the mevalonate pathway with vitamin cofactors, and while meat and fish are the richest dietary sources, it is also present in plant-based foods such as vegetable oils, nuts, and whole grains [123,124,125,126]. Its reduced form, ubiquinol, prevents lipid peroxidation and protects proteins and DNA by quenching lipid radicals. It is capable of regenerating α tocopherol by reducing the tocopheroxyl radical back to its active form [126]. Vitamin E refers to a group of fat-soluble compounds (tocopherols and tocotrienols), with α-tocopherol being the most biologically active. It neutralizes lipid peroxidation chain reactions by donating a hydrogen atom to lipid peroxyl radicals, forming relatively unreactive α tocopheroxyl radicals. Vitamin E acts as an antioxidant in cell membranes and is supported by glutathione peroxidase, the activity of which depends on selenium intake [127]. While vitamin E is found in plants, higher intake is necessary when polyunsaturated fatty acids are present in the diet. Polyphenols, which are synthesized by plants in response to stress, also function as antioxidants and help protect cells from oxidative stress [128].

Table 1 presents the minimum recommended doses of selected antioxidants and vitamins according to FEDIAF (Nutritional Guidelines) [129] in commercial dog food, alongside the selected antioxidant and vitamin dosages used in various interventional studies in adult and aged dogs.

B-complex vitamins (e.g., B1, B2, B3, B5, B6, B9, B12) were included in Table 1 due to their indirect and direct (B6) [139] role in oxidative stress and antioxidant defense [140]. They act as coenzymes in mitochondrial energy metabolism by supporting enzymes in the Krebs cycle and in the electron transport chain, thus indirectly preventing excessive ROS formation, as they are not direct antioxidants because they do not chemically neutralise free radicals. Instead, they reduce electron loss from the ETC, which is one of the main sources of mitochondrial ROS formation. For example, derivatives of the B vitamins thiamine, riboflavin, niacin, pantothenic, and lipoic acid are also crucial for acetyl Coenzyme A supply to the tricarboxylic acid cycle [141] as cofactors for the enzyme pyruvate dehydrogenase, a pyruvate dehydrogenase complex [142]. Thiamine is also important for the function of the α-ketoglutarate dehydrogenase complex, which converts α-ketoglutarate to succinyl- Coenzyme A, reducing another NAD+ molecule and feeding more electrons to the ETC for ATP synthesis [143,144]. Vitamins riboflavin (B2), niacin (B3), B6, B12, folic acid, pantothenic acid, and vitamin C are needed also to synthesize coenzyme [145]. Like most animals, but unlike humans, dogs can synthesize vitamin C in the liver from glucose and therefore do not have a dietary requirement for this vitamin [146]. Nevertheless, vitamin C may become relevant in the context of age-related changes in senior dogs [57]. However, supplementation studies in dogs younger than 2.5 years and older than 7 years have shown no significant effects of vitamin C on antioxidant status or immunologic parameters [132].

Beyond dietary requirements and dosages (Table 1), it is also important to understand the biological roles of different antioxidants and how they influence oxidative balance in dogs. Table 2 provides an overview of key dietary antioxidants, their primary mechanisms of action, and reported effects in canine studies. Together, Table 1 and Table 2 highlight both the quantitative (dose) and qualitative (mechanistic) aspects of antioxidant nutrition in dogs.

In the study by Sechi et al. (2017) [147], a randomized, controlled clinical trial was conducted involving 11 healthy therapy dogs (6 females and 5 males of various breeds, with a mean age of 2.7 ± 0.8 years). The dogs were divided into two groups: one received a commercial diet without antioxidants for 18 weeks, and the other received a diet supplemented with antioxidants. After 18 weeks, a significant reduction in ROS (measured by the derivatives of reactive oxygen metabolites method), triglycerides, and creatinine was observed in the antioxidant-supplemented group, while an increase in amylase was noted in the group fed without antioxidants. Following the crossover of the diets, a decrease in amylase and alanine aminotransferase was observed in both groups. The study concluded that an antioxidant-rich diet improves cellular metabolism and reduces free radical levels in dogs.

In the study by Anturaniemi et al. (2020) [34], dogs with atopic dermatitis fed a raw food diet showed improved innate immunity and reduced oxidative stress, which may help prevent hypersensitivity and immune-related disorders during early development. The study suggests that a raw meat-based diet may reduce the production of ROS in skin cells, leading to less oxidative stress—a key factor in atopic dermatitis. The researchers found that genes regulating immune function and inflammation were more active in dogs fed raw meat, indicating enhanced innate immunity, although chronic inflammation remains undesirable. The authors of another study in atopic dogs receiving vitamin E for 8 weeks achieved similar results. They found clinical improvements (lower canine atopic dermatitis extent and severity index scores and pruritus intensity) in vitamin E group compared to placebo. In addition, in vitamin E group a significant increase in the vitamin E concentration and the antioxidant capacity of lipophilic antioxidants was found, but not the total antioxidant capacity compared to placebo [36].

A randomized, placebo-controlled study investigated the effects of CoQ_10_ supplementation (200 mg/day for 3 months) in dogs with myxomatous mitral valve disease (MMVD). While no significant changes were observed in oxidative stress markers, lymphocyte subpopulations, or clinical and echocardiographic parameters, CoQ_10_ supplementation did result in a decreased neutrophil percentage and increased lymphocyte counts in dogs with congestive heart failure. These findings suggest a potential anti-inflammatory or immunomodulatory effect of CoQ_10_ in advanced stages of MMVD. Overall, the supplementation was safe and well tolerated with no reported adverse effects [148].

The study by Adolphe et al. (2012) [149] examined the impact of complex carbohydrates on oxidative stress in dogs and demonstrated a potential protective effect against oxidative stress associated with hyperglycemia. Peas exhibited the lowest glycemic index (29%) compared to barley and rice (51% and 55%, respectively), though no significant differences in postprandial glucose peaks were observed between the carbohydrate sources. Consumption of glucose solution significantly increased methylglyoxal levels—a byproduct formed during glycolysis from the degradation of glyceraldehyde-3-phosphate and dihydroxyacetone phosphate, as well as from the oxidation of aminoacetone and lipid peroxidation products [150]—compared to complex carbohydrates. The study concluded that complex carbohydrates are more effective in mitigating oxidative stress than diets rich in simple sugars [149].

Oxidative stress is closely linked to the formation of advanced glycation end-products (AGEs), which result from reactions between sugars and proteins. The accumulation of AGEs contributes to oxidative stress by increasing the production of ROS through several mechanisms. AGEs can bind to their receptor, activating intracellular signaling pathways that enhance ROS generation via NADPH oxidase and mitochondrial dysfunction [151,152], and reducing antioxidant defense mechanisms [153]. The study by Jimenez (2021) [152], conducted on plasma samples from wild and domestic canids, investigated the relationship between advanced glycation end-products bound to bovine serum albumin (AGE-BSA) concentrations and body mass or age in domestic dogs, and compared these results with those from wild canids. The findings revealed no correlation between AGE-BSA levels and body size or age in either group. This suggests that AGE formation is an evolutionarily conserved trait in domestic dogs, while oxidative stress levels differ between the two groups. Furthermore, the study indicates that domestic dogs exhibit upregulated lipid metabolism, which may account for differences in lipid damage across lifespan and body size variability.

Oxidative stress and longevity are influenced not only by the nutritional composition of the diet but also by the timing and amount of food intake. Caloric restriction, without malnutrition, has been consistently shown to reduce oxidative damage, improve mitochondrial function, and extend lifespan across multiple species by lowering ROS production and enhancing cellular repair mechanism [154]. Furthermore, meal timing, including intermittent fasting and time-restricted feeding, can modulate circadian rhythms and metabolic pathways, leading to decreased oxidative stress and improved longevity [155,156,157]. These dietary patterns help optimize antioxidant defenses and reduce chronic inflammation, which are critical factors in aging and age-related diseases [155]. Thus, ‘what,’ ‘when,’ and ‘how much’ one eats is crucial determinant of oxidative balance and healthy aging. For example, the study by Bray et al. (2022) [156] found that not only diet composition but also feeding frequency influences the aging process in dogs. A large-scale study involving 24,000 dogs showed that adult dogs fed once daily had, on average, better cognitive performance and a lower risk of gastrointestinal, dental, orthopedic, renal, and hepatic disorders compared to those fed multiple times per day. The impact of caloric intake on canine longevity was further supported by a study conducted by Kealy et al. (2002) [157]. Dogs in the test group received 25% less food throughout their lives than the control group. Results demonstrated that dogs with restricted diets had lower body weight, reduced body fat, and decreased serum levels of triglycerides, triiodothyronine, insulin, and glucose compared to controls. These dogs also exhibited significantly longer median lifespans and a delayed onset of clinical signs of chronic diseases. However, the study did not include data on actual caloric intake, which would help determine whether the observed benefits were due to caloric restriction itself or the reduced feeding frequency.

Ultimately, it should be emphasized that antioxidants may also have non-beneficial, or even harmful effects. An increase in cellular antioxidants could have an effect on redox regulation and signalling transduction, as a basal level of ROS is essential for the maintenance of normal cell function. At low to moderate concentrations, ROS act as important second messengers in various redox-sensitive signalling pathways and influence processes such as cell proliferation, differentiation, autophagy, immune responses and stress adaptation [158,159]. This phenomenon is referred to as redox signalling and plays a crucial role in physiological homeostasis. ROS induce several other biological processes, including a transient increase in intracellular Ca^2+^ concentration, phosphorylation of specific proteins, activation of some other transcription factors, modulation of eicosanoid metabolism and stimulation of cell growth [160]. Conversely, excessive supplementation with exogenous antioxidants can weaken these positive ROS-mediated signals. Studies have shown that high doses of antioxidants such as vitamin C, vitamin E or N-acetylcysteine can suppress adaptive cellular responses, impair exercise-induced mitochondrial biogenesis and even promote tumour growth in certain models by interfering with ROS-dependent signalling processes, apoptosis processes or immune activation [161,162]. This paradoxical effect is referred to as “antioxidant-induced reductive stress” or disruption of redox homeostasis.

There is limited scientific evidence that antioxidant supplementation in dogs can produce no benefit or even negative effects, particularly when given at high doses. For example, in the population of systemically ill hospitalised dogs, combination antioxidant supplementation did not alter redox state or clinical outcome [163]. Additionally, antioxidants from plant extracts and products and vitamins E and C alone were less effective in improving cognition in aging cats and dogs [51].

Taken together, studies consistently show that diet plays a central role in modulating oxidative stress in dogs, with omega-3 fatty acids, antioxidants such as CoQ_10_ and vitamin E, and caloric restriction improving redox balance, immune function, and even longevity. However, results diverge regarding the extent of benefit, as some trials report limited or no effects, and excessive antioxidant supplementation can disrupt physiological redox signaling. Thus, current evidence converges on the idea that nutrition is a powerful modulator of oxidative stress, but its impact depends strongly on nutrient type, dose, and feeding strategy.

## 6. Discussion

Oxidative stress is a significant factor contributing to canine aging and the development of age-related diseases such as cognitive dysfunction, osteoarthritis, cancer, and cardiovascular disorders [25]. The use of antioxidants—such as vitamins C and E, omega-3 fatty acids, plant phenols and CoQ_10_—has proven effective in mitigating the harmful effects of free radicals, thus enhancing overall canine health and contributing to healthier aging.

Research on the impact of oxidative stress on canine aging indicates that it plays a key role in the progression of chronic diseases and cognitive decline in dogs. Oxidative damage to cells caused by free radicals accelerates aging; however, antioxidants serve as a critical mechanism to reduce such damage. Findings from numerous studies, including those by Blanchard et al. (2025) [51], Dowling and Head (2012) [52], and Cotman et al. (2002) [79], have demonstrated that antioxidant-enriched diets can significantly improve cognitive performance and reduce the incidence of chronic disease symptoms in dogs. Additionally, antioxidants have shown substantial benefits in the prevention and alleviation of symptoms related to conditions such as osteoarthritis, atopic dermatitis, heart disease, and cancer, highlighting their broad positive effects on canine health. On the other hand, some studies did not show beneficial effects of antioxidant supplementation [51,57,132,148,163,164].

ROS were initially regarded solely as toxic byproducts of aerobic metabolism, but they are now recognized as crucial regulators of physiological processes including stress responses, pathogen defense, and cellular signaling. An imbalance in favor of antioxidants can lead to so-called “antioxidative stress”, which may also have harmful effects, particularly when synthetic antioxidants are consumed in excessive amounts [165].

Although antioxidants neutralize ROS and can reduce oxidative stress, this is not always beneficial for health. Disruption of the natural balance between ROS and antioxidants may interfere with essential cellular signaling pathways. Overuse of antioxidant supplements and diets may, in fact, be detrimental, as suggested by certain studies linking excessive antioxidant intake with increased mortality. Both extremes—oxidative and antioxidative stress—can result in adverse outcomes. Therefore, maintaining a balanced equilibrium between ROS and antioxidants is essential, as is a careful assessment of oxidative stress levels in the body before recommending antioxidant use [2].

Despite the promising evidence, further research is needed to precisely determine the optimal types and dosages of antioxidants most effective in slowing the aging process. To date, most clinical trials investigating antioxidant supplementation have been relatively short in duration and therefore insufficient to address its potential role in modulating the processes of aging and longevity. Instead, the majority of studies have been limited to monitoring a few markers of oxidative stress and other health indicators, usually assessed in blood samples. Given these limitations, there is a clear need for future long-term clinical trials specifically designed to assess the impact of antioxidant supplementation on clinically meaningful outcomes, including health span and lifespan. An additional limitation is the scarcity of studies specifically addressing these questions in dogs, despite the fact that canine models represent an excellent opportunity to investigate antioxidant interventions in the context of natural aging and age-related diseases. More targeted research in this species would therefore be of particular value. Research in this area represents an important step toward enhancing the quality of life and lifespan in dogs and may contribute to a deeper understanding of the role of nutrition in aging.

### Critical Perspective and Limitations

Despite extensive evidence supporting the potential benefits of antioxidant supplementation in dogs, several important limitations must be acknowledged. Many studies are short-term, involve small sample sizes, or focus on specific breeds under controlled experimental conditions, which limits the generalizability of findings to the broader canine population. Variability in antioxidant type, dosage, and combination across studies further complicates direct comparisons and may partly explain conflicting results.

Additionally, most research emphasizes biomarkers of oxidative stress or cognitive performance rather than long-term, clinically meaningful outcomes such as lifespan, health span, or incidence of age-related diseases. While antioxidants can mitigate oxidative damage, ROS are increasingly recognized as critical for redox signaling, immune function, and stress adaptation. Excessive or prolonged supplementation may therefore disrupt these physiological processes, potentially inducing “antioxidative stress” or reducing adaptive responses.

Nutritional context, including diet composition, caloric intake, and feeding frequency, also modulates antioxidant efficacy, yet these factors are often insufficiently controlled in existing studies. Furthermore, research predominantly involves domestic dogs, whereas wild canids, which exhibit distinct longevity patterns and antioxidant responses, remain underrepresented. Comparative studies could therefore provide valuable insight into species-specific oxidative mechanisms.

Taken together, while antioxidant interventions hold promise for improving health and cognitive function in aging dogs, caution is warranted. Optimal antioxidant types, dosages, and feeding strategies remain unclear, and high-quality, long-term, clinically relevant trials are necessary to establish evidence-based recommendations.

## 7. Conclusions

Oxidative stress is a key driver of canine aging and chronic disease, primarily through damage to lipids, proteins, and DNA. Antioxidants such as vitamins C and E, coenzyme Q10, polyphenols, omega-3 fatty acids, and SAMe show promise in reducing oxidative damage and improving health outcomes in dogs. While current evidence supports the potential benefits of antioxidant-rich diets, study results are variable, and veterinarians should view these strategies as complementary rather than definitive therapies until more robust, long-term clinical trials confirm their efficacy.

## Figures and Tables

**Table 1:** Minimum recommended doses of selected antioxidants and vitamins in commercial dog food and antioxidant and vitamin dosages used in various interventional studies.

Antioxidant/Vitamin	Dog Category	Antioxidant/Vitamin Level	Reference
Selenium	Wet diets - Typical active adult dog - Inactive adult dog	- 23 µg/100 g DM - 27 µg/100 g DM	FEDIAF, 2024 [130]
	Dry diets - Typical active adult dog - Inactive adult dog	- 18 µg/100 g DM - 22 µg/100 g DM	
	Old beagle dogs - control diet - test diet	- 0.59 mg/kg food - 0.53 mg/kg food	Pan et al., 2018 [131]
Vitamin A	- Typical active adult dog - Inactive adult dog	- 606 IU/100 g DM - 702 IU/100 g DM	FEDIAF, 2024 [130]
	Adult beagle dogs, divided into two age groups (<2.5 years and >7 years) - control diet - test (enriched) diet	- 3.81 mg/kg DM - 3.81 mg/kg DM	Hesta et al., 2009 [132]
Vitamin C	Old beagle dogs - control diet - test diet	- 0 mg/kg food - 84.7 mg/kg food	Pan et al., 2018 [131]
	Adult beagle dogs, divided in two age groups (<2.5 years and >7 years) - control diet - test (enriched) diet	- 68.8 mg/kg DM - 68.8 mg/kg DM + 30 or 60 mg vitamin C per day (orally)	Hesta et al., 2009 [132]
	Old dogs (>6 years) - control diet - test (enriched) diet	- 0 ppm (0 mg/kg food) - 559 ppm (559 mg/kg food)	Chapagain et al., 2020 [133]
	Old and young beagle dogs, both receiving. - control diet - test (enriched) diet	- <30 ppm (<30 mg/kg food) - 80 ppm (80 mg/kg food)	Milgram et al., 2004 [134]
	Adult dogs	- 500–1000 mg PO/24 h	McMichael, 2007 [135]
Vitamin D	- Typical active adult dog - Inactive adult dog	- 55.20 IU/100 g DM - 63.90 IU/100 g DM	FEDIAF, 2024 [130]
	Adult beagle dogs, divided in two age groups (<2.5 years and >7 years) - control diet - test (enriched) diet	- 0.0254 mg/kg DM - 0.0254 mg/kg DM	Hesta et al., 2009 [132]
Vitamin E	- Typical active adult dog - Inactive adult dog	- 3.60 IU/100 g DM - 4.17 IU/100 g DM	FEDIAF, 2024 [130]
	Old beagle dogs - control diet - test diet	- 44 mg/kg food - 551 mg/kg food	Pan et al., 2018 [131]
	Adult beagle dogs, divided into two age groups (<2.5 years and >7 years) - control diet - test (enriched) diet	- 11.5 mg/kg DM - 11.5 mg/kg DM	Hesta et al., 2009 [132]
	Old dogs (>6 years) - control diet - test (enriched) diet	- 499 ppm (499 mg/kg food) - 839 ppm (839 mg/kg food)	Chapagain et al., 2020 [133]
	Old and young beagle dogs, both receiving. - control diet - test (enriched) diet	- 120 ppm (120 mg/kg food) - 1050 ppm (1050 mg/kg food)	Milgram et al., 2004 [134]
	Old beagle dogs	Based on weight (PO): - 5.01–10 kg: 67 mg daily - 10.01–15 kg: 100.5 mg daily - 15.01–20 kg: 134 mg daily	Araujo et al., 2008 [136]
	Dogs in general	- 400 IU PO/24 h	McMichael, 2007 [135]
Vitamin B1 (Thiamine)	- Typical active adult dog - Inactive adult dog	- 0.21 mg/100 g DM - 0.25 mg/100 g DM	FEDIAF, 2024 [130]
	Old beagle dogs - control diet - test diet	- 10.39 mg/kg food - 18.67 mg/kg food	Pan et al., 2018 [131]
Vitamin B2 (Riboflavin)	- Typical active adult dog - Inactive adult dog	- 0.60 mg/100 g DM - 0.69 mg/100 g DM	FEDIAF, 2024 [130]
	Old beagle dogs - control diet - test diet	- 13.19 mg/kg food - 13.35 mg/kg food	Pan et al., 2018 [131]
Vitamin B5 (Pantothenic acid)	- Typical active adult dog - Inactive adult dog	- 1.42 mg/100 g DM - 1.64 mg/100 g DM	FEDIAF, 2024 [130]
	Old beagle dogs - control diet - test diet	- 18.57 mg/kg food - 34.07 mg/kg food	Pan et al., 2018 [131]
Vitamin B6 (Pyridoxine)	- Typical active adult dog - Inactive adult dog	- 0.15 mg/100 g DM - 0.17 mg/100 g DM	FEDIAF, 2024 [130]
	Old beagle dogs - control diet - test diet	- 6.11 mg/kg food - 11.05 mg/kg food	Pan et al., 2018 [131]
	Old beagle dogs	Based on weight (PO): - 5.01–10 kg: 41 mg daily - 10.01–15 kg: 61.5 mg daily - 15.01–20 kg: 82 mg daily	Araujo et al., 2008 [136]
Vitamin B12 (Cyanocobalamin)	- Typical active adult dog - Inactive adult dog	- 3.35 µg/100 g DM - 3.87 µg/100 g DM	FEDIAF, 2024 [130]
	Old beagle dogs - control diet - test diet	- 0.053 mg/kg food - 0.100 mg/kg food	Pan et al., 2018 [131]
Vitamin B3 (Niacin)	- Typical active adult dog - Inactive adult dog	- 1.64 mg/100 g DM - 1.89 mg/100 g DM	FEDIAF, 2024 [130]
	Old beagle dogs - control diet - test diet	- 63.45 mg/kg food - 102.57 mg/kg food	Pan et al., 2018 [131]
Vitamin B9 (Folic acid)	- Typical active adult dog - Inactive adult dog	- 25.80 µg/100 g DM - 29.90 µg/100 g DM	FEDIAF^,^ 2024 [130]
	Old beagle dogs - control diet - test diet	- 1.94 mg/kg food - 3.94 mg/kg food	Pan et al., 2018 [131]
S-Adenosyl-L-Methionine (SAMe)	Dogs in general	20 mg/kg BW/day (IV)	McMichael, 2007 [135]
N-acetylcysteine	Dogs in general	50 mg/kg BW	McMichael, 2007 [135]
Ubiquinone (coenzyme Q_10_)	Dogs in general	2 mg/kg BW	McMichael, 2007 [135]
Ubiquinone (coenzyme Q_10_)	Adult beagle dogs	30 mg daily orally	Tomsič et al., 2009 [137]
Ubiquinone (coenzyme Q_10_)	Adult beagle dogs	30 mg daily orally	Prošek et al., 2008 [138]

DM = Dry Matter; PO = Per Os; IU = International Unit; BW = Body Weight; IV = Intravenous. The table presents antioxidant and vitamin levels exactly as reported in the original sources. Units were preserved from the original publications to maintain accuracy. Some values are expressed per 100 g dry matter (DM), which refers to the nutrient content in the food after removing water, while others are expressed per kg of food (as-fed basis), which includes moisture. Because the original studies do not provide sufficient information to convert between these bases reliably, harmonization of units is not possible.

**Table 2:** Overview of key dietary antioxidants, their mechanisms of action, and reported effects in dogs.

Antioxidant/Nutrient	Primary Mechanism of Action	Reported Effects in Dogs
Vitamin E	Lipid-soluble antioxidant; prevents lipid peroxidation in membranes; regenerates via glutathione peroxidase (selenium-dependent)	Improved skin health (atopic dermatitis), reduced oxidative stress markers, and better cognitive function in aged dogs
(α-tocopherol)		
Vitamin C	Water-soluble antioxidant; scavenges ROS in cytosol; regenerates vitamin E	Limited benefit in young dogs; potential support in aged dogs; some studies show no effect
(ascorbic acid)		
Coenzyme Q_10_	Electron carrier in ETC, lipid-soluble antioxidant; stabilizes membranes; regenerates vitamin E; reduces lipid peroxidation	Improved energy metabolism, reduced oxidative damage; mixed results in cardiac disease trials
(ubiquinone/ubiquinol)		
Polyphenols	Scavenge ROS; modulate redox-sensitive signaling pathways; anti-inflammatory	Improved cognition in aged dogs; possible protection in skin and metabolic disorders
(plant-derived)		
Omega-3 fatty acids	Incorporated into membranes; modulate inflammation; improve mitochondrial function	Reduced oxidative stress markers; improved immune response; support in cognitive decline
(EPA, DHA)		
B-vitamins	Cofactors in mitochondrial metabolism; reduce electron leakage from ETC; indirectly lower ROS	Support mitochondrial efficiency; improved antioxidant enzyme activity; indirect role in oxidative balance
(B1, B2, B3, B6, B9, B12)		
S-Adenosyl-L-methionine (SAMe)	Methyl donor; supports glutathione synthesis	Improved hepatic function; enhanced antioxidant capacity

DHA = docosahexaenoic acid; EPA = eicosapentaenoic acid; ETC = the electron transport chain; ROS = Reactive Oxygen Species.

## Data Availability

No new data were created or analyzed in this study.

## Abbreviations

The following abbreviations are used in this manuscript:

ROS	Reactive oxygen species
NADPH	Nicotinamide adenine dinucleotide phosphate
DNA	Deoxyribonucleic acid
SOD	Superoxide dismutase
CoQ_10_	Coenzyme Q_10_
IGF-1	Insulin-like growth factor 1
DHA	Docosahexaenoic acid
ATP	Adenosine triphosphate
ETC	Electron transport chain
EPA	Eicosapentaenoic acid
MDA	Malondialdehyde
MMVD	Myxomatous mitral valve disease
AGEs	Advanced glycation end-products
AGE-BSA	Advanced glycation end-products bound to bovine serum albumin
